# Lack of effects of simvastatin on smoking cessation in humans: A double-blind, randomized, placebo-controlled clinical study

**DOI:** 10.1038/s41598-018-21819-7

**Published:** 2018-03-01

**Authors:** Isabelle Ingrand, Marcello Solinas, Pierre Ingrand, Emilie Dugast, Pierre-Jean Saulnier, Marie-Christine Pérault-Pochat, Claire Lafay-Chebassier

**Affiliations:** 1 INSERM, Clinical Investigation Center CIC 1402, University of Poitiers, CHU Poitiers, Poitiers, France; 2 Department of Epidemiology & Biostatistics, Faculty of Medicine, Poitiers, France; 3 INSERM U-1084, Experimental and Clinical Neurosciences Laboratory, University of Poitiers, Poitiers, France; 4 Department of Clinical Pharmacology, Poitiers University Hospital, Poitiers, France

## Abstract

A recent pre-clinical study has shown that brain-penetrating statins can reduce risks of relapse to cocaine and nicotine addiction in rats. Based on this information, we conducted a randomized, double-blind, placebo-controlled, proof-of-concept trial to assess the efficacy of simvastatin in smoking cessation. After informed consent, 118 participants received behavioral cessation support and were randomly assigned to a 3-month treatment with simvastatin or placebo. The primary outcome was biochemically verified abstinence or smoking reduction at 3-month post-target quit date (TQD). Secondary outcomes were abstinence during weeks 9–12 post-TQD, prolonged abstinence or reduction at months 6 and 12 post-TQD, safety and craving assessed at each visit during the 3-month period of treatment. Simvastatin treatment was not associated with higher 3-month abstinence or smoking reduction compared to placebo. There was no significant difference in any of the secondary outcomes. Simvastatin was well tolerated. Over 3 and 9 months follow-up period, 78% simvastatin and 69% placebo participants were retained in the study. At 6 and 12 months, smoking remained significantly reduced from baseline in both groups. Our results demonstrate that a 3-month simvastatin treatment (40 mg/day), added to individual behavioral cessation support, does not improve significantly smoking cessation compared to placebo in humans.

## Introduction

According to the World Health Organization, smoking is the largest preventable cause of disease and death in the world^1^. Although the prevalence of every-day tobacco use has dropped in most nations since 1990, the total number of smokers has increased^2^. In 2015, 6.4 million deaths worldwide were attributable to smoking, representing a 4.7% increase in smoking-attributable deaths since 2005. This number is likely to reach 8 to 10 millions a year by 2030^1,2^. The benefits of smoking cessation have been clearly proven in terms of morbidity and mortality for different diseases related to tobacco, especially for lung cancer^3,4^. Although over 70% of smokers want to quit, less than 5% of quit attempts are successful annually^5^.

Currently, there are only 3 first-line approved medications in the USA and Europe for smoking-cessation: nicotine replacement therapy (NRT), sustained release bupropion, and varenicline, which are widely recommended in many national guidelines. Nevertheless, the therapeutic effectiveness of NRT is relatively modest, bupropion is not widely used because of its safety profile and varenicline’s use is limited because of fear of potential cardiovascular or neuropsychiatric adverse effects^6,7^. Therefore, the discovery of new medications that could facilitate abstinence and reduce relapse to cigarette use represents a pressing necessity to reduce risks associated with tobacco smoking.

We recently reported in rats that brain-penetrating statins - simvastatin and atorvastatin - can reduce risks of relapse to addiction^8^. In fact, chronic treatment with low doses of statins daily during a 21-day period of abstinence, significantly reduced cocaine or nicotine seeking compared with placebo without altering seeking for food. Based on this information, we hypothesized that simvastatin could have beneficial effects on smoking cessation in humans. Therefore, we conducted a randomized, prospective, double-blind, placebo-controlled, proof-of-concept trial of the efficacy of simvastatin (with behavioural counseling) for smoking cessation. The primary objective of the study was to determine whether simvastatin would increase abstinence or reduce tobacco smoking at the end of 3-month medication treatment compared to placebo. Secondary objectives included evaluation of the continuous abstinence rate for weeks 9–12 post-target quit date (post-TQD), nicotine withdrawal symptoms during medication treatment and prolonged abstinence or reduction at months 6 and 12 post-TQD.

## Results

**Figure 1:**
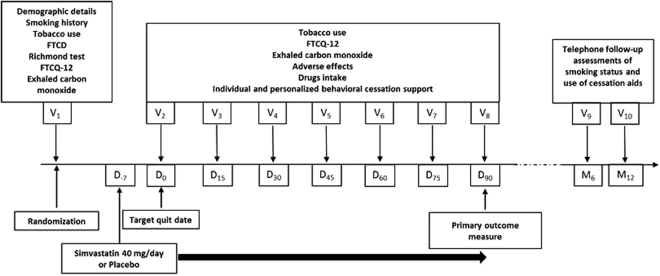
Study procedures. At baseline (visit 1 V_1_), participants were randomized to receive either 40 mg orally once a day simvastatin or matched placebo for 3 months in addition to individual and personalized behavioral cessation support. The Target Quit Date (TQD, Day_0_) occurred after a 7-day medication induction phase. After TQD, participants in each group were prospectively reviewed every two weeks for up to the end of the treatment phase (Day_90_). In addition, two follow-up interviews were telephonically performed at month 6 (M_6_) and month 12 (M_12_).

**Figure 2:**
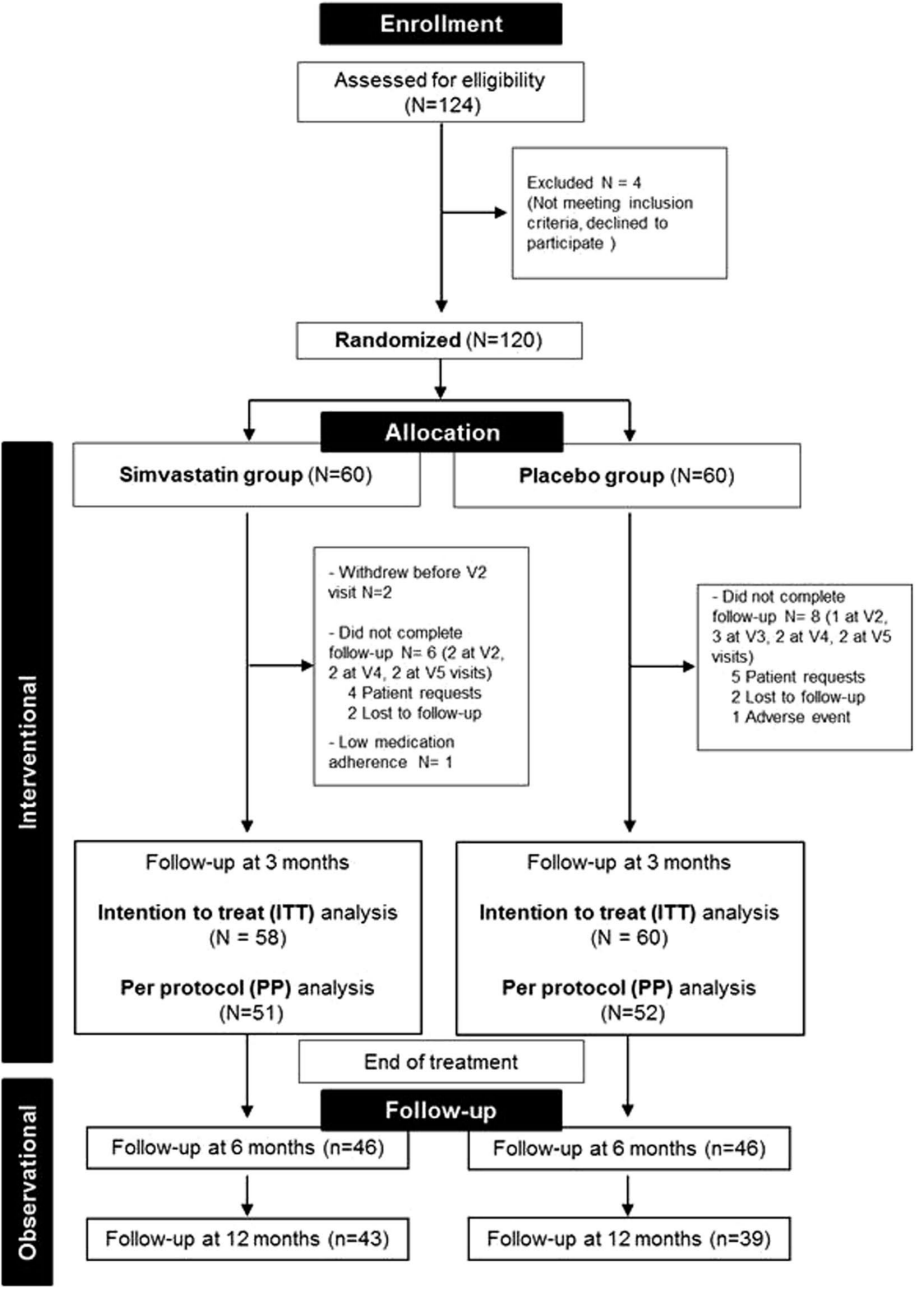
Flowchart of participants through the trial.

Study procedures are presented in Fig. 1 and flowchart of participants through the trial in Fig. 2. Of 120 participants who underwent randomization, two patients in the group simvastatin withdrew their consent (Fig. 2). Overall, 103 (86%) participants completed the 3-month follow-up.

### Baseline Characteristics

Participants in the simvastatin group (n = 58) and the placebo group (n = 60) did not differ significantly at baseline according to demographic and smoking-related variables (Table 1). However, the number of female participants and of children smokers in the home was lower in the placebo group (47% vs 35%, p = 0.063; 17% vs 5%, p = 0.086 in the simvastatin group compared to the control group, respectively). The baseline daily consumption of cigarettes was slightly higher in the simvastatin group (22.4 ± 8.9 vs 21.5 ± 10.9, in the simvastatin group compared to the control group, respectively; p = 0.23), however the exhaled carbon monoxide concentration was not different (42.3 ± 20.5 ppm vs 42.3 ± 19.4 ppm; p = 0.84). According to the Fagerström score, participants in the two randomization groups had similar mild dependence score (5.4 vs 5.3 in the simvastatin and in the placebo group respectively; p = 0.79). As evidenced by the mean FTCQ-12 (French Tobacco Craving Questionnaire-12) total score, at baseline participants had low craving scores and these scores were significantly higher in the simvastatin group (4.05 ± 1.05 vs 3.70 ± 0.95; p = 0.036).

**Table 1:** Baseline demographic and smoking characteristics of the participants.

	Simvastatin n = 58	Placebo n = 60	*p value*
Age (year)	44.3 ± 10.4	42.6 ± 10.7	0.44^a^
Male sex	31 (53%)	39 (65%)	0.063^b^
Weight (kg)	72.5 ± 16.8	71.9 ± 16.7	0.73^a^
Marital status			0.85^b^
Single	22 (38%)	21 (35%)	
Married/Cohabiting	36 (62%)	39 (65%)	
Other smokers in the home			
Partner (n = 75)	18 (50%)	22 (56%)	0.65^b^
Children	10 (17%)	3 (5%)	0.086^b^
Education			0.89^b^
Junior high school	19 (33%)	22 (37%)	
Senior high school	18 (32%)	20 (33%)	
Undergraduate or higher	20 (35%)	18 (30%)	
Working	47 (81%)	51 (85%)	0.63^b^
Number of cigarettes per day	22.4 ± 8.9	21.5 ± 10.9	0.23^a^
Years of smoking	28.4 ± 10.4	26.1 ± 10.6	0.34^a^
Number of previous quit attempts	2.4 ± 1.7	2.4 ± 1.5	0.76^a^
Years since the last quit attempt	2.9 [1.3;8.5]	3.5 [1.8;7.4]	0.70^a^
Motivation score	7.8 ± 1.4	8.0 ± 1.4	0.33^a^
Fagerström Test for Cigarette Dependence score	5.4 ± 2.2	5.3 ± 2.5	0.79^a^
Exhaled carbon monoxide (ppm)	42.3 ± 20.5	42.3 ± 19.4	0.84^a^
French Tobacco Craving Questionnaire-12 items score	4.05 ± 1.05	3.70 ± 0.95	0.036^a^

Notes: Data are means ± SD or median [IQR] or n (%). ^a^Wilcoxon-Mann-Whitney test. ^b^Fisher exact test.

### Smoking Outcomes

Regarding intention-to-treat (ITT) analysis, simvastatin was not associated with higher 7-day point-prevalence abstinence or smoking reduction ≥50% rates at 3 months. At this time point, abstinent participants were 14% in the simvastatin group and 22% in the placebo group and participants who reduced their consumption by 50% or more were 46% in the simvastatin group and 45% in the placebo group (p = 0.30) (Table 2). When considering per protocol (PP) strategy, the analysis yielded essentially similar results, 16% of abstinent participants at 3 months with simvastatin vs 25% with placebo (p = 0.16).

**Table 2:** Smoking reduction and abstinence by treatment groups. Notes: Data are means ± SD or n (%) [95% Confident interval], TQD target quit date.

	Simvastatin n = 58	Placebo n = 60	*p value*
**3 months post-TQD**			
Primary outcome			
100% Abstinence*	8 (14%) [6; 25]	13 (22%) [12; 34]	0.30^a^
50%–99% Reduction*	27 (46%) [33; 60]	27 (45%) [32; 58]	
<50% Reduction*	23 (40%) [27; 53]	20 (33%) [22; 47]	
Weeks 9–12 post-TQD abstinence rate*	8 (14%) [6; 25]	12 (20%) [11; 32]	0.46^b^
% of reduction	64.7 ± 30.5^d^	69.9 ± 28.4^d^	0.39^c^
**6 months post-TQD**			
100% Abstinence*	8 (14%)	14 (23%)	0.56^a^
50%-99% Reduction*	16 (28%)	11 (18%)	
<50% Reduction*	34 (59%)	35 (58%)	
% of reduction	53.1 ± 37.3^d^	54.1 ± 49.1^d^	0.52^c^
**12 months post-TQD**			
100% Abstinence*	8 (14%)	9 (15%)	0.99^a^
50%-99% Reduction*	11 (19%)	10 (17%)	
<50% Reduction*	39 (67%)	41 (68%)	
% of reduction	40.5 ± 42.4^d^	43.5 ± 48.3^d^	0.68^c^

*Validated as exhaled air carbon monoxide concentration ≤8 ppm ^a^Cochran-Armitage exact trend test. ^b^Fisher exact test. ^c^Wilcoxon-Mann-Whitney test. ^d^Wilcoxon test for matched pairs p < 0.0001.

**Figure 3:**
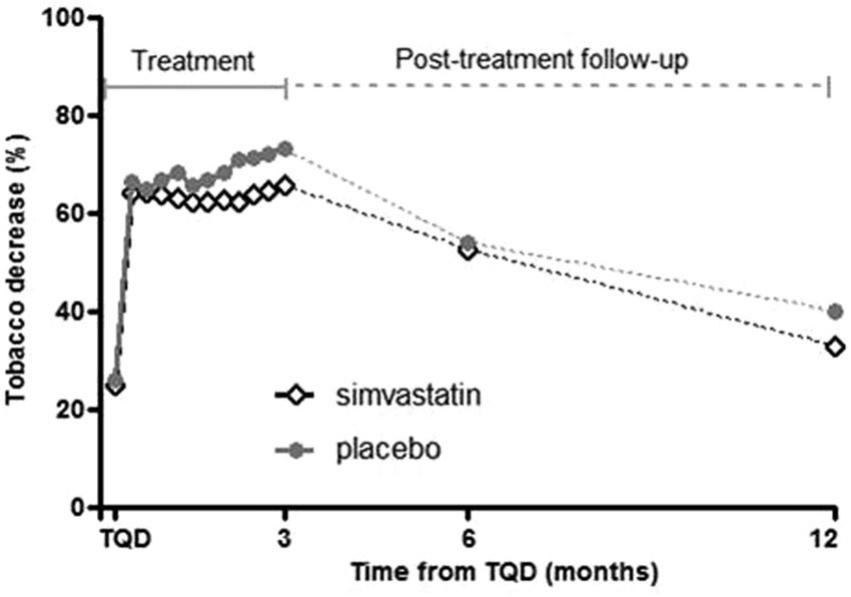
Decrease in tobacco smoking over the treatment and follow-up periods. Over the 3 months treatment period, there was a reduction of cigarettes per day but this reduction was similar in simvastatin- and placebo-treated groups. During the follow-up period, smoking increased similarly in both groups at 6 months and at 12 months, but the reduction remained significant from baseline (p < 0.0001 for both treatments).

Additionally, a comparison of simvastatin and placebo groups for rates of sustained abstinence at weeks 9–12 post-TQD (14% vs 20%), revealed no significant differences (p = 0.46). The percentage of reduction in weekly cigarette consumption from baseline to month 3 was high in the two groups and did not differ between simvastatin and placebo groups (64.7 ± 30.5% vs 69.9 ± 28.4%, p = 0.39). Concerning the decrease in tobacco consumption over 3 months, ANOVA of the percent of reduction of cigarettes per day revealed no treatment effect (p = 0.41) but a significant main effect for time (p < 0.0001), showing that both groups improved from baseline to month 3, with a significant decrease that appeared as soon as the first week following the treatment initiation (p < 0.0001 for both treatments Fig. 3).

### Tobacco craving

**Figure 4:**
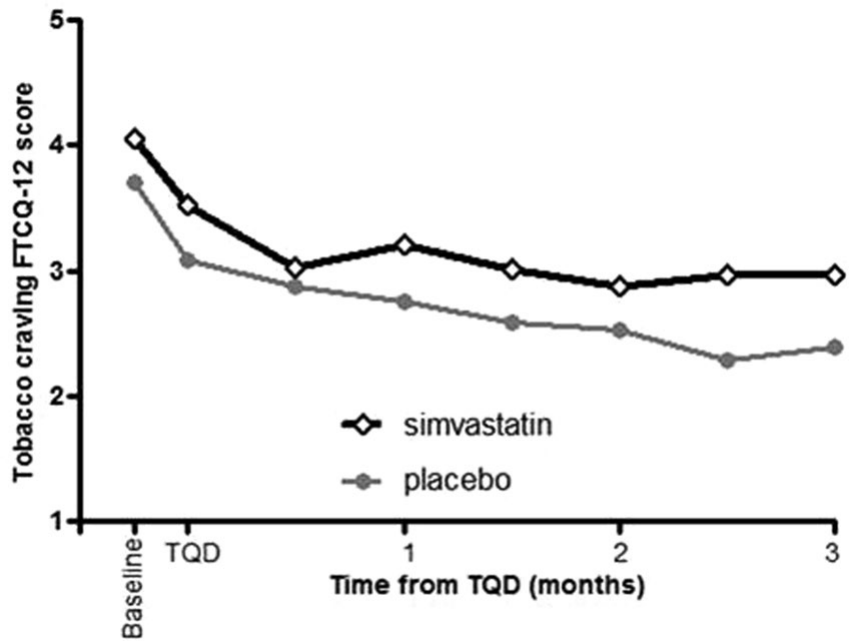
Evolution of craving assessed by FTCQ-12 over the treatment period. Over the 3 months period of treatment, a significant and progressive decrease in craving over time (p < 0.0001 for time effect) was observed in both groups. The mean FTCQ-12 score remained moderately higher (p = 0.038) in the simvastatin group vs placebo.

Figure 4 shows evolution of craving assessed by FTCQ-12. A significant and progressive decrease over time (p < 0.0001 for time effect) was observed in both groups with significant pairwise comparisons until 3 months post-TQD. Repeated measures ANOVA over time shows that the mean FTCQ-12 score remained moderately higher (p = 0.038) in the simvastatin group vs placebo.

### Safety

Simvastatin was well tolerated, and the only two patients who reported serious adverse events not related to the research (a rupture of the Achilles tendon during a football game declared at third visit, and a cataract surgery and myopia of the left eye, declared at the final visit), were in placebo group. As shown in Table 3, adverse events (AEs) related to the experimental drug occurred in 43 participants (74%) in the simvastatin and in 43 participants (72%) in the placebo group (p = 0.40). The various non-serious AEs related to simvastatin or placebo that were observed after randomization by more than 5% of participants in at least one study arm are reported in Table 3. Headache, insomnia/abnormal dreams, muscle pain, smoking aversion, nausea/vomiting, diarrhea, abdominal pain, coated tongue, mood disorders, dysgueusia and constipation were the most frequently reported events in both groups. Body weight increase was not different between simvastatin and placebo groups + 1.4 ± 1.0 vs + 1.3 ± 0.4 respectively (p = 0.75). There was a significant AST increase between baseline and 3 months in simvastatin group (17.4 ± 4.7 UI/L vs 19.8 ± 4.7 UI/L; p = 0.0011) as well as in placebo group (18.2 ± 6.6 UI/L vs 20.2 ± 7.5 UI/L; p = 0.020) but this increase was not different between groups (p = 0.56). For ALT, a significant increase occurred in simvastatin group (18.7 ± 8.4 UI/L vs 22.8 ± 9.3 UI/L; p = 0.0028), and not in placebo group (18.9 ± 8.3 UI/L vs 20.5 ± 10.5 UI/L, p = 0.55). Nevertheless the AST and ALT values remained within the normal range. No significant difference was observed for CPK in any groups (113.4 ± 46.8 UI/L vs 116.1 ± 55.0 UI/L and 110.0 ± 48.1 UI/L vs 103.9 ± 38.5 UI/L in simvastatin group and in placebo group, respectively).

**Table 3:** Adverse events (AEs) related to the experimental drug.

Number of patients presenting AEs related to the experimental drug	Simvastatin (n = 58)	Placebo (n = 60)
0 AE	15 (26%)	17 (28%)
1 AE	24 (41%)	14 (23%)
2 AEs	10 (17%)	16 (27%)
3 AEs	5 (9%)	10 (17%)
4 AEs	4 (7%)	3 (5%)
AEs related to investigational drug*		
Headache	18	16
Insomnia/ Abnormal dreams	11	13
Muscle pain	6	4
Smoking aversion	9	6
Nausea/ Vomiting	4	9
Diarrhea	3	4
Abdominal pain	6	5
Coated tongue	4	3
Mood disorders	3	4
Dysgueusia	3	6
Constipation	1	3

*Non-serious AEs related to simvastatin or placebo that were observed after randomization by more than 5% of participants in at least one study arm.

### Prolonged abstinence

92 participants (78%) completed the follow-up interview at 6 months (46 simvastatin and 46 placebo) and 82 (69%) at 12 months (43 simvastatin and 39 placebo). Total abstinence at 6 months was maintained at 18.6% rate (simvastatin 14%, placebo 23%, Table 2) then decreased moderately but not significantly in both groups at 12 months (simvastatin 14%, placebo 15%, Table 2). Smoking increased similarly in both groups at 6 months and moreover at 12 months, but the reduction remained significant from baseline (p < 0.0001 for both treatments, Fig. 3).

## Discussion

The main finding of this study is that a 3-month treatment with simvastatin does not improve self-reported abstinence, the number of cigarettes smoked, continuous abstinence and reduction in craving compared to placebo. Therefore, despite the promising pre-clinical background supporting the effectiveness of using brain-penetrating statins in management of smoking cessation^8^, we failed to show a benefit of simvastatin compared to placebo. Whereas these results may be seen as somewhat disappointing, it should be noticed that, unfortunately, it is not uncommon that results obtained in animal models of psychiatric disorders are not replicated in humans. For example, fenofibrate, another approved lipid-lowering drug, was found to be effective in reducing nicotine addiction-related effects^9^ but it failed to decrease smoking in dependent smokers^10^. In the same line, notwithstanding a large amount of data in rodents supporting its role in addiction and relapse^11^, recent studies in humans have failed to demonstrate anti-craving effects of corticotropin releasing factor (CRF) compounds in humans^12^. Notwithstanding these failures, it is important to keep comparing animal and human findings and to publish even negative results. This may (1) lead to a better awareness of the limitations, at least in term of predictive validity, of the current animal models, (2) shed lights into differences between humans and rodents that can explain different effects; and finally (3) improve and refine current animal models.

Reasons for the lack of simvastatin efficacy for smoking cessation can be multifaceted. First, it is possible that the brain penetration of simvastatin in human patients was poor and insufficient. In our preclinical experiments, reduction in drug seeking was observed with administration of brain-penetrating statins, simvastatin and atorvastatin whereas administration of pravastatin, a statin with low brain penetrability, did not^8^. Whereas simvastatin appears to have good brain penetrability in rats^13,14^, species-specific physiological differences may exist between rodents and humans that can influence the efficacy of the drug. As a matter of fact, it has been shown that whereas simvastatin has positive effects in rodent and monkey models of Parkinson’s disease, these effects do not appear to translate to humans^15^. The 40 mg/day dose used in Tison’s^15^ study as in ours is the most widely used dose for lipid control. Whereas it is possible that higher doses and other regimens of administration may be effective for smoking cessation, it should be noticed that the risk of adverse effects of statins increases with increasing doses and such treatments could be difficult to implement in the general population. Secondly, it is possible that the behavioral support provided in this study produces significant effects that may have masked the effects of statins. In fact, in our study, intervention combined pharmacotherapy and individual behavioral counseling. Research nurses, trained by smoking cessation medical experts and applied this counseling in accordance to a standardized operational procedure, were in charge of providing the individual and personalized help and support to enrolled patients. This intensity of the behavioral support was substantially greater than what is typically provided, with repeated sessions every two weeks, which might create a stronger placebo response. There is high-quality evidence that individually-delivered smoking cessation counseling can assist smokers to quit^16^ and that counseling associated with medication can more than double the success rate^17^. In our study, the abstinence rate after three months of treatment was 18% that is in accordance with the literature. Success rates of smoking cessation treatments (5-months-after-treatment) range between 8.5% (minimal or no counseling or self-help) and 27.6% (intense counseling and medication), depending on contact time and intensity, number and length of sessions, number and type of clinicians involved and number and type of counseling formats and interventions^17^. This kind of medical management may also explain the results of this trial suggesting a seemingly higher quit rate compared to some of the other studies in the literature. The drop-out rate at 23% and 31% at 6 and 12 months follow-up is lower in comparison to the 6-month 60–70% reported in the literature^18^. Moreover, at 6- and 12-month follow-up, abstinence or smoking reduction were maintained for almost of participants. A recent systematic review and meta-analysis reported that average abstinence rates (6 months or more) were 17%, 19%, 27% and 31% for NRT, bupropion, varenicline and combination NRT, respectively^19,20^. These results show that face-to-face treatment may result in higher commitment of the patients and suggest that more efforts should be made into implementing this kind of treatment as a first choice in the management of tobacco addiction.

An interesting aspect of this study is that, in our population, statins did not produce more adverse effects than placebo. It has been claimed that statin therapy increases the rates of many types of adverse event, including muscle pain or weakness and liver disease. The idea that so-called “statin intolerance” is a common problem has been widely publicized not just in the medical literature but also in the media, which can lead to under-use of statins among individuals at increased risk of cardiovascular events^21^. Our current study found absolutely no differences in simvastatin vs placebo on safety what is in accordance with large-scale evidence from randomized controlled trials showing that almost all of the symptomatic adverse events that are attributed to statin therapy in routine practice are not actually caused by it^22^.

## Conclusion

In conclusion, our findings do not support beneficial effects of simvastatin (40 mg/day) for smoking cessation. On the other hand, the high rates of smoking reduction obtained in both groups in this study highlight the benefit of frequent behavioral counseling in obtaining drastic and long lasting reductions in smoking. Given the high health costs associated with tobacco smoking, these results suggest that more efforts should be made to implement these safe and relatively inexpensive behavioral approaches in the general population.

## Methods

### Study design

A randomized, parallel-group, double blinded, placebo-controlled clinical trial was conducted at the Clinical Investigation Center of the University Hospital of Poitiers (France) between April 2015 and November 2016 (ClinicalTrials.gov NCT02399709, registered on March 26, 2015, https://clinicaltrials.gov/ct2/show/NCT02399709). The study consisted of a 3-month active treatment period with a 9-month follow-up. The study design was approved by the French Regional Ethics Committee (January 06, 2015 n°2014-004978-42) and Drug Regulatory Agency (February 25, 2015 n°141558A-32). All methods were performed in accordance with the relevant guidelines and regulations. All participants in the study gave their informed written consent and received financial compensation for their participation.

### Study participants

Eligible men and women aged 18–70 years were recruited throughout fliers and posters, newspaper and radio advertising. Inclusion criteria were daily smoking ≥10 cigarettes/day for at least 1 year and being motivated to quit. Exclusion criteria were contraindication to simvastatin, active treatment by lipid-lowering agent, history of mental disorder (depression, psychosis, cognitive disorder, mental retardation), substance misuse or alcohol dependence, ≥3-months cigarette smoking abstinence in the previous year, use of smoking-cessation medication (NRT, bupropion, varenicline) or undergoing cognitive-behavioral therapy or use of clonidine or nortriptyline or electronic cigarette for smoking cessation in the last 3 months, pregnancy, breast-feeding and women of childbearing potential without adequate method of contraception.

### Study procedures

After a telephone screening procedure, eligible participants were scheduled for a collective information visit at the clinical investigation center. After they signed the informed consent form, participants underwent an in-person screening assessment (Fig. 1). At this baseline visit, participants were randomly 1:1 assigned to receive either 40 mg orally once a day in the evening simvastatin or matched placebo for 3 months in addition to individual and personalized behavioral cessation support. Smoking cessation counseling was based on French guidelines^23,24^. Simvastatin and matched placebo tablets were provided by the same pharmacist of the Poitiers University Hospital along the trial in indiscernible packages.

The TQD was decided with participants when they received the treatment and were asked to start their treatment one week before TQD. After TQD, participants attended every two weeks an on-site visit. The first and last visits were performed by investigators whereas other visits were performed by trained research nurses who were in charge of providing the individual and personalized support and encouragement to enrolled patients. In addition two follow-up interviews were telephonically performed at month 6 and month 12.

### Data collection

At baseline, data were collected on demographic, medical history, smoking history, cigarette dependence severity (with the Fagerström Test for Cigarette Dependence (FTCD)^25^) and motivation to quit (with the Richmond test^26^). Participants also received a diary in which to record their daily drug intake and daily number of cigarettes.

At each study visit, we recorded data on craving (with the FTCQ-12items^27^), exhaled carbon monoxide concentration (MicroCO®, Eolys, Lyon, France), body weight and all adverse events. Self-reported treatment compliance (total number capsules taken) was monitored. Blood samples were collected for safety evaluation: liver function (aspartate transaminase [AST], alanine aminotransferase [ALT]) and creatinine phosphokinase [CPK] at first and final visits and urine sample for pregnancy test for premenopausal patients.

Participants were contacted at months 6 and 12 post-TQD for blinded telephone follow-up assessments of smoking status and use of cessation aids. Participants were encouraged to complete all study visits even if treatment was discontinued and/or they had failed maintaining abstinence.

### Randomization

Randomization was performed by the methodologist with a computer-generated table (SAS version 9.3, SAS Institute, Cary, NC) using random block sizes. Inclusions were performed sequentially. All investigators who enrolled and assigned participant to intervention and participants remained blinded to the randomization process until the end of the study.

### Outcome measures

The primary outcome was self-reported abstinence or smoking reduction ≥50% during the week before the 3-month follow-up visit. Participants were considered abstinent if they self-reported abstinence for 7 days before the assessment and provided a breath sample with a carbon monoxide concentration of less than 8 ppm.

The secondary outcomes measures included (1) continuous abstinence rate for weeks 9–12 post-TQD with biological validation (exhaled carbon monoxide concentration ≤8 ppm), (2) score of the FTCQ-12, (3) incidence of adverse effects and (4) self-reported prolonged abstinence at months 6 and 12-post-TQD.

Participants who failed to provide a breath sample or provided a breath sample during the interventional part of the study (3 months post-TQD) with a CO level of >8 ppm were considered as non-abstinent smokers. Participants who discontinue before the theoretical end of study (3 months post-TQD) were considered as smokers.

### Statistical analysis

We calculated that based on a 25% expected detectable difference in efficacy on smoking reduction or abstinence between simvastatin and placebo, 80% power was achieved with 50 evaluable patients in each group (20% placebo vs 45% simvastatin, one-sided 5% exact Fisher test^28^). Data validation and database freeze were done prior to unblinding. All randomized participants except those who left the study prematurely (before visit 2, one week after initiation of treatment), were included in the ITT analysis. Participants who left before the theoretical end of study were excluded from the PP analysis and, in ITT analysis, were considered to have continued tobacco consumption and therefore not abstinent from their study outcome and at 3 months.

All statistical analyses were performed using SAS version 9.4 (SAS Institute, Cary, NC). Items of FTCQ-12 were rated on a 7-item Likert scale (strongly disagree to strongly agree).The FTCQ-12 general craving score was derived by summing scores of each items then dividing by the total number of items (raw scores on 4 reverse-keyed items were inverted, Berlin I. *et al*.^27^). Descriptive statistics were presented as mean ± standard deviation (SD) or median with interquartile range for continuous variables and as number (percentage) for categorical variables. Baseline socio-demographic and smoking characteristics between groups were compared using Wilcoxon-Mann-Whitney test for continuous variables or Fisher exact test for categorical variable.

The primary outcome was compared by the exact version of the Cochran-Armitage trend test. The 95% confidence intervals were calculated using the exact method. The secondary outcomes were compared by an exact Fisher test or a non-parametric Mann-Whitney test. The intra-group differences were tested with the Wilcoxon test for matched pairs. The significance level of the tests was 5% (two-sided). Repeated measures mixed analysis of variance (ANOVA) with unstructured covariance matrix was used to estimate the differences from baseline in tobacco consumption and tobacco craving according to treatment across study visits.

### Data availability

The datasets generated during and/or analysed during the current study are available from the corresponding author on reasonable request.

## Electronic supplementary material

CONSORT 2010 Checklist

ADDICSTATINE-Protocole

Avis ANSM

Amendement CPP
