# Supplementary material for: Lack of effects of simvastatin on smoking cessation in humans: A double-blind, randomized, placebo-controlled clinical study
Source: Sci Rep. 2018 Mar 1;8:3836. doi: 10.1038/s41598-018-21819-7 (PMC5832803; doi:10.1038/s41598-018-21819-7)
Supplement: Supplementary file 2 — ADDICSTATINE-Protocole [file 41598_2018_21819_MOESM2_ESM.docx]

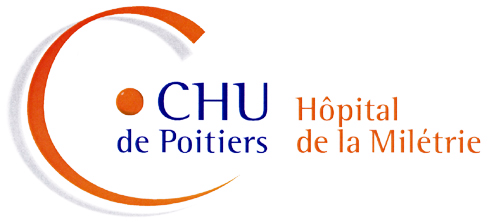


**ADDICSTATINE: Pilot randomized double blind, placebo controlled trial of the efficacy of simvastatin for smoking cessation**

Version n° 6, 04/06/2015

**RESPONSIBLE PARTY :**

Centre Hospitalier Universitaire de Poitiers

2 rue de la Milétrie

BP 577

86021 POITIERS cedex

Tél : 05.49.44.46 65

Fax : 05.49.44.30.58

**PRINCIPAL INVESTIGATOR**

Pr Marie-Christine PERAULT-POCHAT

Centre Hospitalier Universitaire de Poitiers

Service de Pharmacologie clinique et Vigilances

2 rue de la Milétrie- BP 577 - 86021 Poitiers cedex

Tél : 05 49 44 44 53 / Fax : 05 49 44 38 45

E-mail : [m.c.perault-pochat@chu-poitiers.fr](mailto:m.c.perault-pochat@chu-poitiers.fr)

**METHODOLOGIST**

Pr Pierre INGRAND

Unité d’Epidémiologie, de Biostatistique et Registre des Cancers

INSERM CIC-P 802

Faculté de Médecine et de Pharmacie

Centre Hospitalier Universitaire de Poitiers

2 rue de la Milétrie - 86021 Poitiers cedex

Tél : 05 49 45 43 45

E-mail : [pierre.ingrand@univ-poitiers.fr](mailto:pierre.ingrand@univ-poitiers.fr)

| RESPONSIBLE PARTY : | **Centre Hospitalier Universitaire de Poitiers**  2 rue de la Milétrie-BP 577  86 021 Poitiers cedex  Tél : 05.49.44.46.65  Fax : 05.49.44.30.58 |
| --- | --- |
|  | |
| CODE TRIAL | **ADDICSTATINE** |
| N° EudraCT | 2014-004978-42 |
| PRODUCT NAME | Simvastatin Mylan® |
| TITLE | Pilot randomized double blind, placebo controlled trial of the efficacy of simvastatin for smoking cessation |
| CLINICAL PHASE | II |
| INDICATION(S) | Smoking cessation |
| PRINCIPAL INVESTIGATOR | Pr PERAULT-POCHAT Marie-Christine  Centre Hospitalier Universitaire de Poitiers  Service de Pharmacologie clinique et Vigilances  2 rue de la Milétrie- BP 577 - 86021 Poitiers cedex  Tél : 05 49 44 44 53 / Fax : 05 49 44 38 45  E-mail : [m.c.perault-pochat@chu-poitiers.fr](mailto:m.c.perault-pochat@chu-poitiers.fr) |
| CPP | Approuvé le 06/01/2015  Par le Comité de Protection des Personnes Ouest III |
| ANSM | Date d’autorisation : 25/02/2015  N° d’autorisation : 141558A-32 |

**CE DOCUMENT CONFIDENTIEL EST LA PROPRIETE DU CHU DE POITIERS.**

**AUCUNE INFORMATION NON PUBLIEE FIGURANT DANS CE DOCUMENT NE PEUT ETRE DIVULGUEE SANS AUTORISATION ECRITE PREALABLE DU CHU DE POITIERS.**

**SYNOPSIS**

| Title | Pilot randomized double blind, placebo controlled trial of the efficacy of simvastatin for smoking cessation |
| --- | --- |
| Responsible party | Centre Hospitalier Universitaire de Poitiers  2 rue de la Milétrie - BP 577  86021 Poitiers cedex  Tél : 05.49.44.46.65  Télécopie : 05.49.44.30.58 |
| Investigator | Pr PERAULT-POCHAT Marie-Christine  Centre Hospitalier Universitaire de Poitiers  Service de Pharmacologie Clinique et Vigilance  2 rue de la Milétrie- BP 577 - 86021 Poitiers cedex  Tél : 05 49 44 44 53 / Fax : 05 49 44 38 45  E-mail : [m.c.perault-pochat@chu-poitiers.fr](mailto:m.c.perault-pochat@chu-poitiers.fr) |
| Context | Tobacco smoking is the number one preventable cause of disease worldwide. Unfortunately, there are few smoking cessation agents and their effectiveness has been shown to be relatively limited and is associated with potential unwanted effects. Therefore, the discovery of new medications that can facilitate abstinence and reduce relapse to cigarette use represents a pressing necessity. In the attempt to find molecules that could reduce drug-seeking behavior, we discovered that simvastatin reduces cocaine and nicotine, but not food, seeking behavior in rats. This discovery of a new therapeutic application for an already marketed class of compounds may greatly facilitate the translation from preclinical to clinical setting. In this project, we aim at investigating whether simvastatin is an effective smoking cessation agent in humans. |
| Main Objective | To evaluate the efficacy after a 3 months treatment by simvastatin versus placebo as an aid to quit smoking. The efficacy is estimated according the tobacco status: no reduction, reduction of 50% or more, abstinence. |
| Secondary Objectives | - To evaluate smoking abstinence rate. A participant is considered abstinent if he/she reports continuous abstinence from smoking confirmed by expired air carbon monoxide ≤8ppm and urinary cotinine concentration ≤10ng/mL during the last month of the 3 months treatment period.  - To evaluate the decrease in cigarette consumption over the 3-month period of treatment with simvastatin versus placebo  - To evaluate the duration of the abstinence from tobacco smoking over the 3-month period  - To evaluate changes in tobacco smoking behavior (craving) |
| Primary end point | Self-reported number of cigarettes smoked after 3 months of simvastatin treatment |
| Secondary end points | - Self-reported continuous abstinence during the last 4 weeks of the 3 months of treatment with simvastatin versus placebo  - Expired air carbon monoxide ≤ 8 ppm  - Urinary cotinine concentration ≤ 10 ng/mL  - Nicotine craving assessed by the FTCQ-12 |
| Study design | Controlled, randomized, double blind 2 parallel groups, clinical trial with an intention-to-treat analysis. |
| Principal inclusion Critèria | - Age >=18 and <=70 years  - Smoking more than 10 cigarettes per day for at least 1 year  - Motivated to quit smoking  - Without legal tutors or subordination  - Affiliated to a health insurance system as required by the French law on biomedical research  - Written informed consent for participation in the study |
| principal exclusion Critèria | - Age < 18 or > 70 years  - Presenting a contraindication to simvastatin use - With depression and/or psychosis and/or cognitive disorder and/or mental retardation or chronic use of medications for these disorders - Substance use disorder other than smoking  - More than 3 months of abstinence from cigarette smoking in the previous year  - Use of nicotine replacement therapy, bupropion, varenicline on last 3 months  - Use of clonidine or nortriptyline on last 3 months  - Undergoing on last 3 months’ cognitive-behavioral therapy for smoking cessation  - Premenopausal women without contraception |
| Arms | Simvastatin (40 mg/day) during 3 months *versus* placebo |
| Number of Patients | 120 smokers : 60 receiving simvastatin and 60 receiving placebo |

**SIGNATURES**

Signing of investigator

| J'ai lu l’ensemble des pages du protocole n°6 du 06/04/2015 de l’essai clinique dont le CHU de Poitiers est le promoteur. Je confirme qu'il contient toutes les informations nécessaires à la conduite de l’essai. Je m'engage à réaliser l’essai en respectant le protocole et les termes et conditions qui y sont définis. Je m'engage à réaliser l’essai en respectant :   - les principes de la “Déclaration d’Helsinki”, - les règles et recommandations de bonnes pratiques cliniques internationales (ICH-E6) et française (règles de bonnes pratiques cliniques pour les recherches biomédicales portant sur des médicaments à usage humain - décisions du 24 novembre 2006), - la législation nationale et la réglementation relative aux essais cliniques, - la conformité avec la Directive Essais Cliniques de l’UE [2001/20/EC] dont une copie de chaque m'a été remise par le promoteur.   Je m'engage également à ce que les investigateurs et les autres membres qualifiés de mon équipe aient accès aux copies de ce protocole et des documents relatifs à la conduite de l’essai pour leur permettre de travailler dans le respect des dispositions figurant dans ces documents. | |
| --- | --- |
| **NOM: Pr PERAULT-POCHAT**  . | Date : 06/03/2015 |

Signing of responsible party

| **Promoteur :** | |
| --- | --- |
| **NOM** : Mr. Jean-Pierre DEWITTE  Pour le Directeur Général et par délégation  Le Directeur de la Recherche  **Aurélien DELAS** | Date : 06/03/2015 |
