# Supplementary material for: Lack of effects of simvastatin on smoking cessation in humans: A double-blind, randomized, placebo-controlled clinical study
Source: Sci Rep. 2018 Mar 1;8:3836. doi: 10.1038/s41598-018-21819-7 (PMC5832803; doi:10.1038/s41598-018-21819-7)
Supplement: Supplementary file 3 — Avis ANSM [file 41598_2018_21819_MOESM3_ESM.pdf]

ansm
Agence nationale de sécurité du médicament et des produits de santé

AUTORISATION D'ESSAI CLINIQUE DE MEDICAMENT A USAGE HUMAIN

Nombre de pages : 1
(incluant la page de garde)

Envoi par Télécopie

Date : 25 FEV. 2015

**Identifiants de l'essai clinique**					
Titre	Pilot randomized double blind, placebo controlled trial of the efficacy of simvastatin for smoking cessation				
Promoteur	CHU DE POITIERS			Réf. CPP	Non disponible
Réf. Promoteur	ADDICTSTATINE	N° EudraCT	2014-004978-42	Réf. ANSM	**141558A-32**

**Expéditeur**	**Destinataire** (demandeur : nom / société / tél.)
ANSM / Direction Produit NEURHO / Equipe DOLORH<br><br>Dossier suivi par : Marie COULON<br>Tél : 33 (0) 1 55 87 38 71.<br>Fax : 33 (0) 1 55 87 33 09	FANNY ABRIAT<br>CHU DE POITIERS<br>0549443796<br><br>Fax **05 49 44 30 58**

**CPP** destinataire en copie	**Ouest III (Poitiers)**	**Fax**	**05.49.46.12.62**	

Vu le code de la santé publique et notamment ses articles L. 1123-8, L. 1123-12 et vu le dossier de demande d'autorisation d'essai clinique adressé à l'Agence nationale de sécurité du médicament et des produits de santé (ANSM) ;

Vu les compléments versés par le promoteur en date du 13 février 2015 et notamment le protocole de l'essai cité en objet modifié (version 4 datée du 10/02/2015), suite à la demande de l'ANSM ;

L'autorisation mentionnée à l'article L. 1123-8 du code de la santé publique est accordée pour l'essai clinique cité en objet. Cette autorisation est valable pour toute la durée de l'essai à compter de la date de la présente décision.

Toutefois, conformément à l'article R. 1123-33 du code de la santé publique, la présente autorisation devient caduque si la recherche n'a pas débuté dans un délai d'un an.

Le directeur
Direction des médicaments en neurologie, psychiatrie, antalgie, rhumatologie, pneumologie, ORL, ophtalmologie, stupéfiants

Philippe VELLA

Je vous demande de transmettre toute demande d'informations complémentaires concernant ce dossier par courriel adressé à la boite : aec-essaiscliniques@ansm.sante.fr . Je vous précise qu'il vous est possible d'utiliser à cet effet le système de messagerie électronique sécurisée Eudralink. Lors de l'envoi de ces dossiers, je vous demande de veiller à reporter dans l'objet du message les mentions suivantes :
- pour les MS transmises à l'ANSM pour information : **MSI/ Réf ANSM du dossier Direction Produit NEURHO / Equipe DOLORH**
- pour les MS soumises pour autorisation ou pour les dossiers mixtes (comportant des modifications soumises pour autorisation et d'autres pour information) : **MSA/ Réf ANSM du dossier Direction Produit NEURHO / Equipe DOLORH**

Si vous ne recevez pas toutes les pages de cette télécopie, veuillez contacter le secrétariat de la Direction Produit NEURHO / Equipe DOLORH au : 33 (0) 1 55 87 30 75.

Confidentialité
Cette transmission est à l'attention exclusive du(des) destinataires ci-dessus mentionné(s) et peut contenir des informations privilégiées et/ou confidentielles. Si vous n'êtes pas le destinataire voulu ou une personne mandatée pour lui remettre cette transmission, vous avez reçu ce document par erreur et toute utilisation, révélation, copie ou communication de son contenu est interdite. Si vous avez reçu cette transmission par erreur, veuillez nous en informer par téléphone immédiatement et nous retourner le message original par courrier. Merci.

Confidentiality
This transmission is intended to the addressee(s) listed above only and may contain preferential or/and confidential information. If you are not the intended recipient, you are hereby notified that you have received the document by mistake and any use, disclosure, copying or communication of the content of this transmission is prohibited. If you have received this transmission by mistake, please call us immediately and return the original message by mail. Thank you.
