# Supplementary material for: Lack of effects of simvastatin on smoking cessation in humans: A double-blind, randomized, placebo-controlled clinical study
Source: Sci Rep. 2018 Mar 1;8:3836. doi: 10.1038/s41598-018-21819-7 (PMC5832803; doi:10.1038/s41598-018-21819-7)
Supplement: Supplementary file 4 — Amendement CPP [file 41598_2018_21819_MOESM4_ESM.pdf]

Comité de Protection des Personnes

OUEST III

Agréé par arrêté ministériel en date du 31 mai 2012,
Constitué selon l'arrêté du Directeur Général de l'ARS Poitou Charentes en date du 25 juin 2012.
C.H.U. La Milétrie
Pavillon le Blaye – Porte 9
2 rue de la milétrie - BP 577 - 86021 POITIERS CEDEX
Tel : 05.49.45.21.57
Fax : 05.49.46.12.62
E-mail : cpp-ouest3@chu-poitiers.fr

DIRECTION RECHERCHE
02 JUIL. 2015
C.H.U. de POITIERS
2015-470

Monsieur Aurélien DELAS
Direction de la Recherche
CHU de Poitiers

Poitiers, le 24 juin 2015

Objet : Modification substantielle n°3
Référence Comité : Protocole n°14.12.46

Monsieur le Directeur,

Le Comité a étudié lors de la réunion du 24 juin 2015 de la modification substantielle n°3, concernant le projet de protocole suivant :

Numéro de l'essai : ADDICTSTATINE

Titre de l'essai : **« Essai randomisé évaluant, en double insu, l'efficacité d'un traitement par la simvastatine versus placebo dans l'aide à l'arrêt de la consommation de tabac »**

Identité du promoteur : CHU de Poitiers – 2 rue de la milétrie – 86021 POITIERS Cedex

Identité de l'investigateur : Pr PERAULT-POCHAT - Service de Pharmacologie Clinique et vigilance
CHU – 2 rue de la milétrie – 80621 POITIERS Cedex

N° identification : 2014-004978-42
Versions :
Lettre de saisine : 04/06/15
Argumentaire Pr Perault-Pochat : 04/06/15
Formulaire de MS : 04/06/15
Formulaire de demande d'avis : 04/06/15
Tableau comparatif : version n°5 du 06/03/15 versus version n°6 du 04/06/15
Protocole : version n°6 du 04/06/15
Résumé : version n°4 du 04/06/15
Note d'information patient + formulaire de consentement : version n°1 du 04/06/15
Liste investigateurs : n°3 du 04/06/15

Objet :
Prolongation de la participation du patient à 12 mois pour connaitre le statut tabagique après 3 et 9 mois d'arrêt de la simvastatine grâce à un contact téléphonique. La durée de la période d'inclusion est de 9 mois. La durée de la participation pour chaque patient est de 12 mois. La durée – partie patient 21 mois et partie analyse 12 mois porte la durée totale de l'étude à 33 mois, avec une date de début au 01/04/2015. Le contact téléphonique à 3 et 9 mois après la V8 est à l'origine d'une nouvelle note d'information patient.

Ajout d'investigateurs avec le Dr Xavier Lemercier, le Dr Jacques Marin, le Dr Geneviève Coulombier, et le Dr Béatrice Bareth dont les CV, n°d'inscription à l'ordre des médecins et accords sont joints.

Le quorum général étant constaté,

Mr	L.	LACOSTE	Qualifié en matière de recherche biomédicale	(T)
Mme	N.	RABAN	Qualifiée en matière de recherche biomédicale	(S)
Mr	D.	DESSEAUVE	Epidémiologiste	(T)
Mr	J.N.	RICHER	Médecin Généraliste	(S)
Mme	M.	CHABIN	Pharmacien	(T)
Mme	M.	AUMOND-SIMONIN	Infirmière	(T)
Mr	P.	THOMAS	Qualifié en matière d'éthique	(T)
Mr	M.	BILLE	Qualifié dans le domaine social	(T)
Mme	F.	BLET	Qualifiée en matière juridique	(T)
Mme	A.	RANGER	Qualifiée en matière juridique	(S)
Mme	V.	BONNAUD	Psychologue	(T)
Mr	D.	MAROUBY	Représentant d'association de malades	(T)

Après avoir entendu le rapporteur, les membres du CPP ont délibéré et émis l'avis suivant :

Le Comité émet un avis favorable pour cette modification substantielle

Soyez assuré, Monsieur le Directeur, de mes sentiments les meilleurs.

Le Vice-président
Dr L. LACOSTE
